# National centre for healthy ageing data platform: Developing a core set of research data from hospital electronic health record systems: A modified Delphi approach

**DOI:** 10.1177/18333583251352310

**Published:** 2025-07-23

**Authors:** Emily Mann, Kim Naude, Tanya Ravipati, Richard Beare, Velandai Srikanth, Nadine E Andrew

**Affiliations:** 1 National Centre for Healthy Ageing, Frankston, Victoria, Australia; 2 Peninsula Clinical School, School of Translational Medicine, Monash University, Frankston, Victoria, Australia; 3 Developmental Imaging, Murdoch Children’s Research Institute, Melbourne, Victoria, Australia; 4 Academic Unit, Frankston Hospital, Peninsula Health, Melbourne, Australia

**Keywords:** health services, electronic health records, data sharing, registries, methodology, health information management

## Abstract

**Background:** Hospital electronic health record (EHR) systems contain extensive data of varying quality and suitability for research. Identifying research suitable data from hospital systems is rarely performed using a structured approach. **Objective:** To define a core set of research suitable data, from multiple hospital datasets and systems for the National Centre for Healthy Ageing Data Platform. **Method:** Candidate data were identified based on: published literature, researcher survey and quality audit. We recruited researchers and clinicians to participate in a modified Delphi method to achieve consensus on the core data. Live online surveys were conducted across 11 datasets. Participants rated the relevance of each data item using a Likert scale and provide additional feedback. Acceptance criteria were set at >70% agreement. Items meeting this threshold were included in the core dataset. **Results:** The end user group contained 16 university and 17 clinician researchers. An initial 144 data items were presented for consideration. Experts provided additional details for specialised items. Consensus to include an item was reached after one (*n* = 127) or two (*n* = 3) rounds with an additional three items identified by end-users. The final core dataset included 133 items across all 11 datasets. **Conclusion:** Our approach proved effective in reaching consensus among diverse researchers, ensuring that the selected data items are relevant for a range of purposes. **Implications for health information management practice:** Having a core dataset will support targeted activities that ensure these items are accurate, readily accessible and used appropriately to support patient care, administrative tasks and research.

## Introduction

Hospital electronic health records (EHRs) contain vast amounts of rich data, with potential to enhance clinical and health service research using data captured during routine clinical care. However, hospital EHR systems are examples of relational databases, with patient data held in multiple siloed datasets, presenting significant challenges in utilising these data comprehensively across datasets and over time (Andrew et al., 2023; Wang et al., 2018). Hospital EHR data are not primarily collected for research. Hence, the quality and completeness of these data vary widely depending on the purpose for which they are collected, the perceived importance from a clinical or operational perspective (e.g. funding) and the systems within which they are documented (Kahn et al., 2016). There is also considerable variability between EHR datasets making it difficult to make assumptions across different EHR systems. This variability makes it difficult for researchers to determine which data items are of sufficient quality for research purposes, and the potential biases that may be introduced by including certain data items in their analyses (Gianfrancesco and Goldstein, 2021; Rusanov et al., 2014).

The sheer volume of EHR data within a health service makes it unfeasible to assess the quality and research utility of every single data item systematically. Researchers or data managers are required to sift through large datasets to identify reliable and relevant information, which can be a time-consuming and resource-intensive process (Hripcsak and Albers, 2013). Moreover, data are generally provided to researchers with a waiver of patient consent making it unethical for health services to release excessively large amounts of data that may not be relevant to answering the research aims (Ritchie, 2017). Consequently, research typically focuses on data from a single unit, system or component of a health service, leaving out potentially valuable insights that could be gained from a more integrated and systematic approach (Fleuren et al., 2021; Johnson et al., 2015; Pfaff et al., 2022).

The challenges of integrating EHR data are further compounded by the heterogeneous nature of data collected from different hospital units, such as intensive care, pharmacy or admission departments (Rusanov et al., 2014). Each unit may have its unique data collection methods, standards and terminologies, further complicating data harmonisation and analysis. Hence, there are few examples where data have been brought together from multiple systems within a health service, highlighting a significant gap in the current research landscape (St Sauver et al., 2012).

At the National Centre for Healthy Ageing (NCHA; Victoria, Australia), we have sought to address many of these challenges, using a consensus-based approach to systematically bring together high-value research data items from multiple datasets held within a health service EHR system. The aim of this study is to describe the steps undertaken to develop a set of core data items for clinical and health service research within a health service EHR system.

## Methodology

### Setting

The NCHA is a partnership between Monash University and Peninsula Health, located in the Frankston/Mornington Peninsula region of Victoria, Australia. This partnership leverages the strengths of both organisations with the overarching objective of transforming health and care related to ageing for all Australians. The NCHA focuses on driving innovation and developing solutions to tackle challenges at individual, community and system levels with a goal to enable Australians to live healthy and meaningful lives aligned with their values and needs.

Monash University is a globally recognised leader in medical research, particularly in the areas of innovative and translational research involving sensitive data. The university’s researchers benefit from significant investments in state-of-the-art infrastructure, platforms and services, which supported the development of the Healthy Ageing Data Platform.

Peninsula Health is the sole public health provider for the Frankston/Mornington Peninsula region, serving a population of approximately 311,000 people. It is also an early adopter of EHR systems. The region, which encompasses both metropolitan and rural areas, has a diverse socio-economic profile, an ageing population (20% aged over 65) and the Mornington Peninsula local government area is ranked second for the number of persons estimated with dementia in 2025 (Dementia Australia, 2023). Residents access the majority of their healthcare through Peninsula Health, which includes two acute care hospitals, two rehabilitation hospitals, and over 10 outpatient and community health centres. Unlike most health services in Australia, Peninsula Health assigns a single unit record (UR) number to patients across all services, allowing data from multiple electronic systems to be linked together. These features made Peninsula Health an ideal partner in the development of a linked EHR derived cohort for ageing-related health services research.

### Stage 1: Data item selection

To manage the extensive and varied data within the Peninsula Health EHR system, a decision was made to prioritise high-value, high-quality data items that are frequently utilised in healthcare research. An existing published review of international literature was identified and used as a reference to identify EHR data items commonly employed in research studies (Bruland et al., 2016). Data dictionaries containing data items that are part of mandatory reporting to the Victorian Health Department, and released to researchers for approved projects by the Victorian Centre for Data Linkage, were also reviewed. While the primary goal of the NCHA is to support transformative research and translation in the field of healthy ageing, the core data items were not required to be exclusively focused on ageing research. This allows the Healthy Ageing Data Platform to be utilised for a wide range of investigator-led research.

#### Data quality audit

An audit of the Peninsula Health EHR systems was conducted in stages prior to each Delphi round, between March 2020 and October 2023 to determine the availability of the data items, and their quality was assessed using the World Health Organisation’s (WHO) Data Quality Review (DQR) framework (World Health Organisation, 2020). Unlike many generic data governance or data management frameworks, the WHO DQR is tailored for health data systems and is specifically built for use in health systems research and epidemiology. It also has a strong focus on de-identified and confidential data, justifying its use over other frameworks. The WHO DQR evaluates data across five key dimensions: (1) accuracy, that is, determining that data represents the real-world context; (2) completeness, the extent to which data fields are devoid of missing or indeterminate values; (3) consistency, comparing data across diverse sources and timeframes; (4) timeliness, assessing if time taken to report data is appropriate for its intended use and (5) integrity, ensuring unauthorised changes to data cannot be made. Four of the five dimensions were measured as part of the data quality audit, with completeness being measured using two criteria: non-missing (not null, not blank; %) and specificity (calculated by excluding unknown, not specified, not stated or “other”; %). Integrity was not measured for the Delphi process but was documented as part of internal processes. During the audit, data items that were not routinely available or scored poorly on multiple aspects of the WHO DQR were excluded.

#### Health service data user survey

A pre-Delphi survey was developed to gather information from health service data users in the hospital (clinical) and university (research) to determine their research interests, types of data previously used/accessed, and the participants perceived quality of these data. In the survey, we also asked about perceived barriers to accessing routinely collected data; such as hospital data, government held or collected data and registry data. The survey instrument consisted of mainly open-ended questions. Questions in the first part of the survey were designed to inform our understanding of participants areas of research, other secondary uses of routinely collected data such as those provided by state or Commonwealth data linkage units and the types of data items they used in their research. The second part of the survey was designed to help us identify negative aspects of participants’ prior experiences, such as the perceived quality and appropriateness of the data for answering their research question, barriers encountered in accessing data and suggestions for how these processes could be improved. The survey was administered through Qualtrics (Version: Oct 2020–2023), a web-based platform for creating and managing surveys.

A descriptive qualitative approach was used to analyse the responses from the surveys. The data were first categorised into key themes based on common topics mentioned by participants. Both qualitative content analysis (to identify recurring themes) and basic descriptive statistics (to count mentions of key issues) were used to summarise the findings.

#### Domain expert consultations

Many of the datasets within the Peninsula Health EHR System have their own data manager. This is especially so for the more specialised datasets such as radiology, pharmacy, costing and mental health. The Data Platform team met with dataset managers to discuss the availability of data items and their suitability for research, data quality and any particular nuances to the datasets that should be considered as part of the Delphi process. Subject matter experts, including experienced researchers specialising in areas, such as radiology, pharmacy, pathology and mental health, were consulted to assist with identifying relevant data items and to provide valuable insights on their utility. Their expertise ensured that data items would be both meaningful and applicable to the specific area of research.

#### Identifying items for review

An exhaustive list of items was collated based on information obtained from the literature and health department reviews, user survey, data quality audit and expert consultations. These data items were then categorised into 11 core datasets based on their research context (e.g. demographics, admissions ) or the specialised dataset in which they were held, resulting in 11 domains: demographic, emergency, inpatient, theatre/surgical, outpatient, pharmacy, pathology, costing, mental health, community health, radiology. Data items that are considered standard for research, such as admission and discharge dates, age, and gender, were classified as “mandatory” and presented but not voted upon during the Delphi workshop. The remaining items were classified as “optional” based on their perceived value for research and presented for voting. Patient identifiers such as name and date of birth were not included in the core datasets for polling.

### Stage 2: Modified Delphi process to obtain consensus on the core data items

#### Recruitment of end user group

University researchers and hospital clinicians from the two NCHA partner organisations were invited to join the end user group and participate in the modified Delphi process to determine core data items. To recruit hospital clinicians, heads of departments and members of the Research Operations Committee were invited to participate or nominate delegates with an interest or experience in utilising health data for research. University researchers were identified through existing research collaborations and suggestion by the heads of Monash University School of Primary and Allied Health Care and Rehabilitation, Ageing and Independent Living. Individuals were invited via email detailing the purpose and time commitment of the process. Once confirmed, participants were sent an information sheet and invited to an online information session about the NCHA Data Platform and Delphi process. Consent to participate, as outlined in the information sheet, was implied by attendance at the information session and subsequent Delphi workshops.

#### Modified Delphi process

The Delphi method, traditionally used for gathering expert opinions, involves multiple rounds of surveys where experts provide feedback, followed by controlled feedback and reconsideration of their responses in subsequent rounds (Dalkey, 1969). Survey rounds are staggered to allow end users to consider their response prior to providing feedback in the second round. The key aspects of a Delphi process are anonymity of responses, iteration (multiple rounds of feedback) and controlled feedback (experts receive a summary of the groups responses and comments). Compared to other methods like Nominal Group Technique or Focus Groups, the Delphi method minimises groupthink by allowing anonymous feedback and offering multiple rounds of input. It also reduces the influence of dominant personalities, fostering more objective decision-making. The iterative nature of the approach is ideal for complex healthcare data, where expert judgment is critical.

The modified Delphi process adapted for this project took place from October 2020 to 2023 and incorporated online meetings and online consensus polling via Zoom. End users were provided with an information pack about the core data items for consideration, prior to attending an online Zoom session. The first round of polling took place via Zoom. Attendees were presented with information about the data item and provided with an opportunity to ask questions and discuss the data item. Data items classified as “mandatory” were presented to the end user group, and an explanation was provided about the necessity to include these items in the core datasets. End users had an opportunity to discuss the data items and provide feedback. Based on discussion without objections, mandatory data items were included in the core datasets without polling by the end user group. Attendees were then asked to rate the “optional” data item for inclusion in the core dataset using a 6-point Likert scale (1. Strongly agree, 2. Agree, 3. Slightly agree, 4. Slightly disagree, 5. Disagree, 6. Strongly disagree). Consensus to include the data item in the core dataset was achieved if ⩾70% of the end user group responded as “agree” or “strongly agree.” End users that could not attend the live Zoom meeting were invited to provide feedback via poll utilising Qualtrics software (Qualtrics, Provo, UT, USA).

Results from the first round of the Delphi process were summarised and provided back to the end user group via email. Individuals had an opportunity to provide additional feedback about the data items that did not reach consensus. This information was provided to the end user group prior to the second round of polling. Items that did not reach a consensus of ⩾70% “agree” or “strongly agree” after the second round of polling were excluded from the core dataset.

### Ethical considerations

Informed consent was implied by attendance at the Delphi workshops. Ethics approval for this work was obtained from the Peninsula Health Office for Research: QA/69403/PH-2020-237290(v2).

## Results

### Stage 1: Data item selection

#### Data identification and quality audit

Our review of the study by Bruland et al (2016), the Centre for Victorian Data Linkage data dictionary and consultation with subject matter experts resulted in 11 source systems being identified as containing research relevant data and the identification of 144 data items commonly utilised in health research across 11 domains. Permission to access these datasets was obtained from the individual dataset managers, and data were drawn for >800,000 registered patients for the quality review.

Data quality varied across data items with those that were part of mandatory reporting to the Health Department having the highest quality rating. From the demographic core dataset completeness (non-missing) ranged from 95% (ethnicity) to 100% (postcode) with a specificity of 65–98%. For inpatient data (January 2016–May 2021), completeness (non-missing) ranged between 98% (discharge method) and 100% (admission type) with a specificity of 93–99%. For Emergency Department attendances (January 2016–May 2021), completeness (non-missing) ranged between 99% (arrival mode) and 100% (visit type), with a specificity of 93% and 100%, whereas items such as tobacco misuse was poorly recorded with low completeness and specificity. A sample of these items are provided in Table 1.

**Table 1.** Sample of data quality dimensions for data items.

Core data items	Accuracy (%)	Completeness		Consistency (%)	Timeliness (%)
		Non-missing (%)^a^	Specificity (%)^b^		
Marital status	99	100	92	98	100
Sex	81	81	98	92	100
Interpreter requirement	100	100	100	100	100
Admission type	76	100	99	100	100
Admission source	95	100	100	95	100
Separation mode	93	99	91	91	100
Arrival mode	91	100	93	89	100
Departure status	97	99	99	86	100

a Not null or blank.

b % calculated by excluding unknown, not specified, not stated or “other.”

#### Health service data user survey

The Health Service Data User Survey was emailed to 24 end users with 16 responding (66.6% response rate). The key areas of research for respondents included Models of Care, Stroke, Falls, Cognition, Dementia, Physical Activity and Health Services. The types of research projects that participants were experienced in included mixed methods (*n* = 13), quantitative (*n* = 15), qualitative (*n* = 13), quality audits (*n* = 6) and clinical trials (*n* = 10).

The majority of participants (*n* = 15; 94%) had experience requesting routinely collected datasets for research or linkage projects. Datasets mentioned included hospital data, registry data, health department data and data from the National Stroke Audit, Ambulance Australia and Australian Institute of Health and Welfare. The types of data items used from these datasets were wide-ranging (See Supplemental material).

Survey responses indicated mixed opinions on whether the data provided was sufficient in scope and quality to be used effectively for research that is, fit for purpose. Several participants mentioned that hospital datasets require extensive cleaning and restructuring before they can be analysed, with data inconsistencies and missing values a recurring issue. One participant wrote: “Health department and hospital data require a lot of cleaning, reorganising and inside knowledge.” According to a few participants, some structured datasets (like registries) were of high quality, but hospital and ambulance data have gaps, such as coding inconsistencies and missing values impacting data reliability and completeness. Many researchers may need to adjust their research questions based on the data they can access rather than designing the ideal study first: “(I) have tended to build the research question around the data we knew we could access rather than the other way around.”

Many of the survey participants (*n* = 12; 75%) experienced difficulties accessing datasets. The main themes emerging from responses included (i) *delays and time constraints*. A significant barrier for nine participants (56.3%) was the extremely long timeframe for receiving data. One participant wrote “It takes 2–3 years to get access to health department data due to lengthy negotiations with the data custodians and waiting for linkages to be done.” (ii) *Administrative and logistical barriers*. According to participants, the approval process for data access can involve significant bureaucracy, including multiple levels of permissions and interactions with data custodians. (iii) *Technical challenges*. Some datasets are incomplete or require multiple requests before the full dataset is obtained. Data extraction processes may also not be well structured: “The process to extract medication administration data was extremely time consuming, with problems including reports ‘timing out’ part way through.” (iv) *Lack of priority for researchers*. A few participants felt that research teams are often not prioritised by hospital data providers or service staff, routine operational reports being given precedence.

### Stage 2: Modified Delphi process to obtain consensus on the core data items

#### Recruitment of end user group

Invitations to join the end user group were sent to 49 individual clinicians and researchers. Of those, 24 accepted, 24 did not respond and 1 declined. The 24 “yes” responders were sent the Health Service Data User Survey (See Supplemental material) and formed the initial end user group. An additional nine individuals, with specific and relevant areas of expertise, joined the end user group throughout the Delphi process. Of the 33 participants (16 university researchers and 17 clinicians) recruited to the end-user group, 15 were female (45%) and the majority (*n* = 28; 93%) held doctorate degrees. All participants that were engaged with the end-user group at the time of each Delphi phase were invited to attend the workshops and provided an information pack. The number of participants engaged in each Phase of Delphi declined over time (Phase 1, *n* = 18; Phase 2, *n* = 14; Phase 3, *n* = 14; Phase 4, *n* = 9; Phase 5, *n* = 6), and the composition of the end-user group was dependent on the core datasets being determined for that phase (See Supplemental material).

#### Modified Delphi process and data item consensus

**Figure 1.**
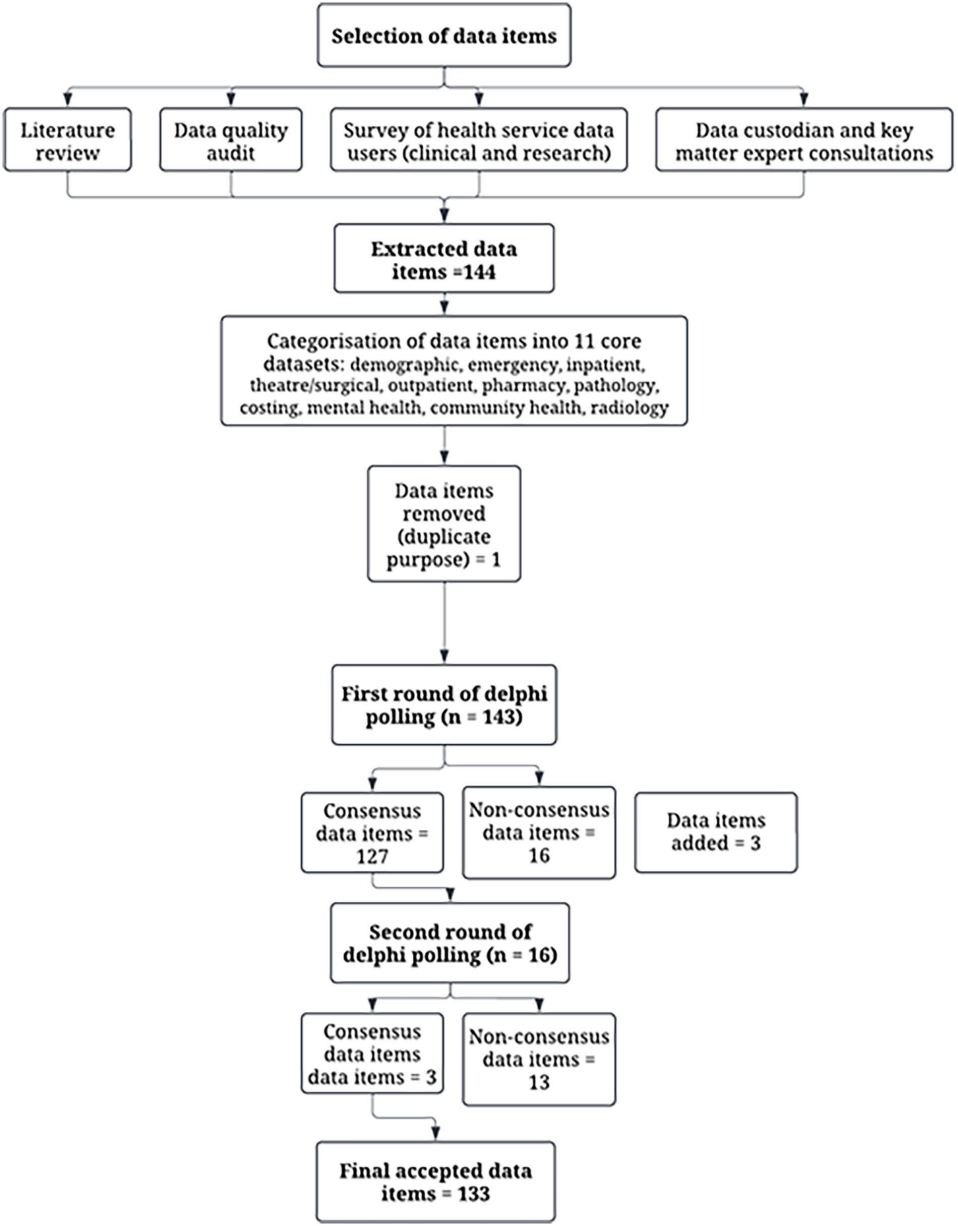
Flowchart of the data item identification and Delphi process.

The 144 initial data items were categorised as “mandatory” or “optional,” based on the research utility and presented to the end user group for discussion and feedback. During discussions, one date item was removed from the list due to duplication and an additional three were added (Figure 1).

Consensus for data items was obtained over five phases of Delphi workshops and surveys. Each phase focussed on the polling of 1–3 core datasets to ensure the end user group had time for discussion about each data item. When required, multiple rounds of polling were conducted at different time points to reach consensus. Phase 1–3 of the process resulted in some data items not reaching consensus in the first round (poll one) and a second round of online workshops or Qualtrics survey was performed and non-consensus items were polled a second time (*n* = 16). All data items reached consensus in the first poll of phases 4 and 5 (Supplemental material). The final list of data items included in each dataset is detailed in Table 2, and details of each core dataset, including those that did not reach consensus, are provided in the Supplemental material.

**Table 2.** Final accepted data items.

Dataset	Final accepted data items
Demographic	*Mandatory:* sex, date of birth, postcode, interpreter requirement
	*Optional:* country of birth, marital status, religion ID, living arrangement, usual accommodation type
Inpatient	*Mandatory:* admission date, primary diagnosis code, other diagnosis code/s, primary procedure code, other procedure code/s, discharge date, died during episode, date of death
	*Optional:* admission type, admitting specialty, admission care type, discharging specialty, discharge destination, intention to re-admit, onset date, duration of stay in critical care unit (CCU), duration of stay in intensive care unit (ICU), transfer reason, systolic blood pressure, diastolic blood pressure, pulse, temperature, height, weight, body mass index, date of vital signs taken, current drug overdose, clinical intervention, tobacco misuse, alcohol misuse, cancer diagnosis date, aged care assessment service status (ACAS), functional independence measure (FIM) score (as assessed on admission), functional independence measure (FIM) score (as assessed at discharge)
Theatre/surgery	*Mandatory:* theatre case specialty, primary procedure code ID, operation type, surgery start date, episode ID, theatre case ID
	*Optional:* unplanned return to theatre, operation outcome, American society of anesthesiologists (ASA) score, anaesthetic type
Emergency	*Mandatory:* arrival date, time to triage, primary diagnosis, additional diagnoses, departure date, triage category
	*Optional:* arrival mode, visit type, departure destination, nature of main injury, injury cause, human intent
Outpatients	*Mandatory:* episode start date, episode program/stream, episode health conditions, episode end date, contact start date, contact program stream, contact professional group, contact purpose, contact inpatient flag, contact duration
	*Optional:* episode other factors affecting health, contact delivery setting
Pharmacy	*Mandatory:* date of medication order, medication order type, medication name, medication dosage, clinician specialty, date of supply, pharmacy identifier, patient identifier
	*Optional:* medication route
Pathology	*Mandatory:* patient type, order date, test name, reason for test, specimen type
	*Optional:* order status, collection date, test result, upper limit, lower limit
Costing	*Mandatory:* encounter type, allied health cost bucket, emergency cost bucket, ward medical cost bucket, ward nursing cost bucket
	*Optional:* critical care unit cost bucket, operating room cost bucket, imaging cost bucket, pathology cost bucket, pharmacy cost bucket, ward supplies cost bucket, prosthesis cost bucket, non-clinical cost bucket
Mental health	*Mandatory:* episode start date, episode end date, program, primary diagnosis
	*Optional:* phase of care, triage outcome, other diagnoses, service contact start date, service contact end date
Community health	*Mandatory:* health condition/s, referral date, referral in provider type
	*Optional:* service stream, service presenting reason, service initial contact date, service end date
Radiology	*Mandatory:* request date, modality, procedure/exam name
	*Optional:* reason for exam, order status, technique, conclusion

#### End-user engagement with the data platform

Following the Delphi process, a number of end user participants requested access to the NCHA data platform dataset, reflecting direct utilisation of the identified data items. Of the 52 use cases, 20 (38%) of these were led by end user participants. The use cases were led by an equal mix of Monash University and Peninsula Health researchers and represented a wide range of project types that included: quality improvement (*n* = 18), healthcare evaluation (*n* = 12), healthcare utilisation (*n* = 8), digital health (*n* = 5), epidemiological and cohort studies (*n* = 5), and clinical trial and interventional studies (*n* = 4). Studies covered areas such as general patient groups for example, people at risk of readmission or attending a specific service, those with social vulnerability for example, homelessness or drug and alcohol problems, or specific clinical groups for example, people with dementia, neurological conditions or diabetes.

## Discussion

In this study, we present a comprehensive and innovative methodology to enhance the understanding and quality of EHR data for use by a diverse cohort of researchers. Utilising a systematic approach to the identification and curation of a core set of EHR data within the broader data ecosystem of a health service has resulted in a core set of data that can be used with confidence by researchers to answer a range of questions relevant to health services research. By understanding researcher needs we were able to identify source systems containing relevant data, gain access to these data through a series of defined activities, build relationships and trust in the data through strong engagement with end users and identify opportunities for data improvement.

Engaging end-users is pivotal for the future success and sustainability of the NCHA data platform, as evidenced by the significant number of use-cases submitted by members of the end-user group. The Delphi process not only facilitated thoughtful and comprehensive decisions in data selection for research purposes but also served as a mechanism for engaging potential users and enhancing transparency within the health service. The collaborative efforts and goodwill of the data owner, Peninsula Health, is vital to the success of the NCHA Data Platform. The Delphi process provided an opportunity for our team to have constructive and focused conversations with individual data managers, which were particularly crucial for accessing and understanding specialised datasets such as pathology and radiology. This also promoted a sense of collaborative ownership over the NCHA Data Platform. For clinicians and researchers, participation in the Delphi process offered a forum to ask questions about the data, consider its potential use for their own research, and suggest additional items relevant to their specific needs.

The activities undertaken to develop our core dataset, although novel in their application to EHR systems, align with those undertaken by Clinical Quality Registries (CQRs). The Australian Framework for National CQRs specifies the systematic identification of data elements that are essential for evaluating and improving healthcare quality (Australian Commission on Safety and Quality in Healthcare, 2024). Like our approach, this typically encompasses a review of relevant literature, an understanding of clinical guidelines or in our case research utility, and expert opinions to prioritise data items (Cadilhac et al., 2016; Wormald et al., 2020). Once the core data elements are identified, it is recommended that they undergo rigorous validation to ensure accuracy, reliability and feasibility of data collection. As with CQRs, our core data items will require ongoing monitoring of utility, frequency of use by researchers and changes in data providence with periodic updates to maintain relevance. Special requests for non-core items will be monitored so that frequently requested non-core items can be considered for inclusion in the core dataset.

Operating principle three of the Framework for National CQRs also recommends data linkage with national and jurisdictional information systems (Australian Commission on Safety and Quality in Healthcare, 2024). Although linkages have been undertaken for a number of CQRs (Andrew et al., 2016; Smith et al., 2024) it has not been feasible to link whole EHR systems for research purposes. Having a core dataset has enabled a recent proof-of-concept linkage of the NCHA core dataset with state and Commonwealth administrative datasets (Andrew et al., 2023).

Having a well-defined core dataset within a linked EHR system has enabled us to establish more robust governance processes than would have been achievable without a core dataset. Data from the NCHA healthy ageing data platform are governed by the “Five Safes” framework, an internationally recognised approach to assess and manage risks associated with data sharing and release. Having a well-defined set of core data has supported compliance with the first three of the “Five Safes” (Ritchie, 2017): (i) safe projects by improving researcher’s ability to communicate their data use to ethics committees, (ii) safe people by providing training and supports specific to the use of EHR core data and (iii) safe data through curation of the core data items to minimise inadvertent identification of individuals. In particular, having a well-defined set of core data has allowed us to provide focussed resources such as detailed data dictionaries, meta data and training webinars for items that end-users have identified as important to their research. Providing resources such as these for an entire data warehouse would not be feasible.

### Limitations

Having a core dataset was both a strength, as outlined above, and a limitation of the NCHA Data Platform, the main limitation being that we were unable to guarantee the quality of non-core data items that could be provided to researchers upon request. Although some quality checking was undertaken as part of the extraction process, the provision of supporting documentation and quality enhancement was not available. Development of the core items was not restricted to items relevant to ageing research and the majority of data items received consensus for inclusion in the core dataset. However, it was restricted to data items that were applicable to a broad range of populations and so did not include some items important for research in specialised groups such as maternity and paediatrics. Although the platform was able to draw upon data from 11 research relevant datasets, including all major clinical information systems, there were a number of clinician-held datasets, generally developed for quality assurance purposes, which were not held within the data warehouse, and we were unable to access these as part of standard procedures. However, we did have capacity to link these internally to a central spine using UR numbers when they were provided to us. While the Delphi process facilitated engagement with research from diverse fields, end users were limited to those associated with the NCHA partner organisations. Additional work is needed to engage external researchers in future plans to release data to researchers from non-partner organisations.

## Conclusion

Our innovative method to enhance the understanding and quality of EHR data for a diverse range of researchers has resulted in the creation of a core set of well-documented, research-grade data derived from various components of a health service EHR system. This core dataset will underpin the ongoing work of the NCHA Data Platform, facilitating semi-automated data extraction, providing metadata to support researcher utilisation and understanding of the data, and establishing a foundational dataset for linkage to other datasets and data types. Notably, we have also provided a blueprint with the potential for scalability to other health services.

## Supplemental Material

sj-docx-1-him-10.1177_18333583251352310 – Supplemental material for National centre for healthy ageing data platform: Developing a core set of research data from hospital electronic health record systems: A modified Delphi approach Supplemental material, sj-docx-1-him-10.1177_18333583251352310 for National centre for healthy ageing data platform: Developing a core set of research data from hospital electronic health record systems: A modified Delphi approach by Emily Mann, Kim Naude, Tanya Ravipati, Richard Beare, Velandai Srikanth and Nadine E Andrew in Health Information Management Journal
