# Supplementary material for: National centre for healthy ageing data platform: Developing a core set of research data from hospital electronic health record systems: A modified Delphi approach
Source: Health Inf Manag. 2025 Jul 23;55(1):100–8. doi: 10.1177/18333583251352310 (PMC12756512; doi:10.1177/18333583251352310)
Supplement: sj-docx-1-him-10.1177_18333583251352310 – Supplemental material for National centre for healthy ageing data platform: Developing a core set of research data from hospital electronic health record systems: A modified Delphi approach [file sj-docx-1-him-10.1177_18333583251352310.docx]

Figure S1, online supplement: NCHA Data Platform End User group survey

Thank you for your interest in participating in the NCHA Data Platform end user group. Prior to the formal Delphi process, we would like to gather some information about how you utilise health data in your research. Please complete the following 5-10 minute survey. Your results will be summarised with other end user group participants and used to guide the core dataset discussion.

Please enter your name below.

___________________________________________________________

Q 1. What are your main areas of research?

________________________________________________________________

Q 2a. What type of research projects do you undertake?

- Qualitative
- Quantitative
- Clinical trials
- Quality Audits
- Mixed methods
- Other

Q 2b. If other, please provide details.

________________________________________________________________

Q 3. Have you requested routinely collected datasets from other groups for research or linkage projects? (i.e. routinely collected data includes data not collected for research purposes, such as hospital data, government held or collected data, and registry data).

________________________________________________________________

Q 4. What type of data items have you used from these datasets?

________________________________________________________________

Q 5. Was the data fit for purpose? (i.e. was it of sufficient scope and quality to be used in the way you intended?)

________________________________________________________________

Q 6a. Have you experienced any difficulties accessing datasets? Please briefly describe.

________________________________________________________________

Q 6b. If yes, what types of datasets were they and do you have suggestions on how this process could be made easier?

________________________________________________________________

Table S1, online supplement: Common data type usage reported by end user group survey participants

| Data Type | Data |
| --- | --- |
| Clinical Data | International Statistical Classification of Diseases and Health Related Problems 10^th^ Revision (ICD-10) codes, therapy occasions of service, diagnoses, disease severity measures, admission/discharge dates, stroke unit care, discharge destination |
| Demographic Data | age, sex, socioeconomic status, rurality, interpreter required, Aboriginal and Torres Strait Islander status, marital status, residential type, state of residence, funding source |
| Outcome Data | readmission within 30 days, mortality, falls, adverse events, hospital readmission data, death data, EQ-5D scores (self-reported health tool) |
| Process Data | therapy duration, care type, processes of care, allied health service input |
| Hospital Admission/Discharge Data | hospital admission dates, discharge dates, emergency department presentation and re-presentation data, ward transfers, nursing home admission |
| Medication/Prescribing Data | drug name, dose, date, location, Pharmaceutical Benefits Scheme (PBS) dispensing items, prescribing data |
| Health Service Utilization | health service use, outpatient appointments, referral to HARP, admission to intensive care unit (ICU) or palliative care |
| Financial/Costing Data | hospital costing data, Medicare items, PBS, Weighted Inlier Equivalent Separation (WIES) & Australian National Subacute and Non-Acute Patient Classification (AN-SNAP) payment data |

Table S2, online supplement: End user group participant details and workshop participation

| Stakeholder No. | Gender | Researcher/Clinician | Research focus | Phase 1 | | Phase 2 | | Phase 3 | | Phase 4 | Phase 5 |
| --- | --- | --- | --- | --- | --- | --- | --- | --- | --- | --- | --- |
|  |  |  |  | Poll 1 | Poll 2 | Poll 1 | Poll 2 | Poll 1 | Poll 2 | Poll 1 | Poll 1 |
| 1 | F | R | Epidemiology | x | x | x | x | x | x | x | x |
| 2 | M | C | Gerontology | x |  |  |  |  |  |  |  |
| 3 | M | R | Allied Health | x | x | x | x | x |  |  |  |
| 4 | M | R | Allied Health | x |  | x | x | x | x |  |  |
| 5 | F | R | Physiotherapy | x | x |  |  |  |  |  |  |
| 6 | F | R | Nursing & midwifery | x | x | x | x | x | x |  |  |
| 7 | F | R | Rehabilitation | x | x | x | x | x | x | x |  |
| 8 | F | R | Addiction | x | x | x | x | x | x | x | x |
| 9 | F | R | Physiotherapy | x | x | x | x | x |  | x |  |
| 10 | M | C | Gerontology | x | x |  |  | x |  |  |  |
| 11 | M | R | Physiotherapy | x | x | x | x | x |  | x | x |
| 12 | F | R | Allied Health | x | x | x | x | x |  |  |  |
| 13* | M | C | Mental Health |  |  |  |  |  |  |  |  |
| 14 | M | C | Pharmacy | x | x | x | x |  |  |  |  |
| 15* | M | C | Medical Imaging |  |  |  |  |  |  |  |  |
| 16 | M | C | Cardiac Imaging | x |  | x | x | x |  |  |  |
| 17* | F | C | Emergency |  |  |  |  |  |  |  |  |
| 18 | M | C | Thoracic Medicine | x |  |  |  |  |  |  |  |
| 19 | M | C | Cardiovascular | x |  |  |  |  |  |  |  |
| 20* | M | C | Gastroenterology |  |  |  |  |  |  |  |  |
| 21 | F | C | Haematology | x | x |  |  |  |  |  |  |
| 22 | M | C | Intensive Care | x | x | x | x |  |  |  |  |
| 23* | F | C | Neurology |  |  |  |  |  |  |  |  |
| 24* | M | C | Respiratory |  |  |  |  |  |  |  |  |
| 25 | F | R | Addiction and Mental Health |  |  | x | x |  |  |  |  |
| 26 | F | C | Pharmacy |  |  | x | x | x | x | x |  |
| 27 | F | R | Health Economy |  |  |  |  | x |  | x |  |
| 28* | F | C | Mental Health |  |  |  |  |  |  |  |  |
| 29 | F | R | Epidemiology |  |  |  |  |  |  | x |  |
| 30 | M | R | Epidemiology |  |  |  |  |  |  | x |  |
| 31 | M | R | Radiology |  |  |  |  |  |  |  | x |
| 32 | M | R | Radiology |  |  |  |  |  |  |  | x |
| 33 | M | C | Medical Imaging |  |  |  |  | x |  |  | x |

**Stakeholders engaged in the Delphi process by providing feedback on potential data items, attending the Delphi workshops and engaging in discussion, but did not participate in the polling.*

Table S3, online supplement: Data item consensus by Core dataset

|  |  | **Poll One** | | **Poll Two** | | **Polling total** | | **Additional data items added** | **Final total** |
| --- | --- | --- | --- | --- | --- | --- | --- | --- | --- |
|  | **Dataset** | **Polled** | **Consensus** | **Re-polled** | **Consensus** | **Consensus** | **Non -Consensus** |  |  |
| Phase 1 | Demographic | 10 | 9 | 1 | 0 | 9 | 1 | 0 | **9** |
|  | Inpatient | 35 | 32 | 3 | 0 | 32 | 3 | 2 | **34** |
|  | Theatre / Surgical | 14 | 10 | 4 | 0 | 10 | 4 | 0 | **10** |
| Phase 2 | Emergency | 12 | 12 | 0 | 0 | 12 | 0 | 0 | **12** |
|  | Outpatient | 12 | 11 | 1 | 1 | 12 | 0 | 0 | **12** |
|  | Pharmacy | 12 | 9 | 3 | 0 | 9 | 3 | 1 | **10** |
| Phase 3 | Pathology | 12 | 8 | 4 | 2 | 10 | 2 | 0 | **10** |
| Phase 4 | Costing | 13 | 13 | 0 | 0 | 13 | 0 | 0 | **13** |
|  | Mental Health | 9 | 9 | 0 | 0 | 9 | 0 | 0 | **9** |
| Phase 5 | Community Health | 7 | 7 | 0 | 0 | 7 | 0 | 0 | **7** |
|  | Radiology | 7 | 7 | 0 | 0 | 7 | 0 | 0 | **7** |
|  |  |  |  |  |  | **130** | **13** | **3** | **133** |

Figure S2, online supplement: Results of the Delphi process for each dataset

Phase 1 (Demographics, Inpatient, Theatre/Surgery)

Within the Demographic dataset, the data item *Religion ID* was not accepted. *Ethnicity (Aboriginal and Torres Strait Islander status)* was polled, however due to its sensitive nature it is not included in the data dictionary. It is available on special request and approval by the Research Office. Within the Inpatient dataset, the data items *Previous Drug Overdose*, *Electrocardiogram (ECG) Completed Date*, and *Laterality of Primary Cancer* were not accepted. The data items *Functional Independence Measure (FIM) Score (as assessed on admission)* and *Functional Independence Measure (FIM) Score (as assessed at discharge)* were added later on based on discussion with members of the end-user group. Within the Theatre/Surgical dataset, the data items *Case Extra Detail ID*, *Source*, *Additional Detail Name*, and *Detail Value* were not accepted. *Surgery Start Date Time* was changed to *Surgery Start Date*.

Phase 2 (Emergency, Outpatient, Pharmacy)

Within the Emergency dataset, the data item *Triage Date* was changed to *Time to Triage*. In the Outpatient Dataset, the data items *Contact Start Date/Time* and *Contact End Date/Time* were changed to *Contact Start Date* and *Contact duration* respectively. The data item *Episode - other factors affecting health* was re-polled in round 2 and accepted. Within the Pharmacy dataset, the data items *Medication order location*, *Medication order status*, and *Dispense from location* were not accepted. The data item *Administration schedule start date* was removed during the first polling workshop as it was deemed through discussion to overlap with the data item *Date of supply* and was therefore not reliable. *Medication Route* was added later on based on discussion with members of the end-user group.

Phase 3 (Pathology)

Within the Pathology dataset, the data items *Collection priority* and *Result unit* were not accepted. The data items *Collection date* and *Lower limit* were re-polled in round 2 and were accepted.

Phase 4 (Costing and Mental Health)

All data items in the Costing and Mental Health datasets were accepted in round 1.

Phase 5 (Community Health and Radiology)

All data items in the Community Health and Radiology datasets were accepted in round 1.
